# Validity, Reliability, and Factor Structure of the Secondary Traumatic Stress Scale-French Version

**DOI:** 10.3389/fpsyt.2019.00191

**Published:** 2019-04-12

**Authors:** Ingo Jacobs, Marion Charmillot, Chantal Martin Soelch, Antje Horsch

**Affiliations:** ^1^Department of Natural Sciences, Medical School Berlin, Berlin, Germany; ^2^Department of Psychology, Sigmund Freud University Berlin, Berlin, Germany; ^3^Department Woman-Mother-Child, Lausanne University Hospital, Lausanne, Switzerland; ^4^Division of Clinical and Health Psychology, University of Fribourg, Fribourg, Switzerland; ^5^Institute of Higher Education and Research in Healthcare, University of Lausanne and Lausanne University Hospital, Lausanne, Switzerland

**Keywords:** STSS, midwives, ESEM, burnout, occupational efforts and rewards, posttraumatic stress, HADS

## Abstract

Secondary traumatic stress (*STS*) is a syndrome including intrusion, avoidance, and arousal due to indirect trauma exposure (e.g., by caring for traumatized patients in a professional context or transgenerational transmission of trauma in familial or cultural systems). Bride et al. (1) developed the Secondary Traumatic Stress Scale (*STSS*), designed to measure these reactions of helping professionals who have experienced traumatic stress through their work with their traumatized clients. This study aimed to validate the French version of the STSS (*STSS-F*) by evaluating factorial and criterion validity. Furthermore, its reliability and other psychometric properties were evaluated. Two-hundred-and-twenty midwives at two university hospitals in the French-speaking part of Switzerland completed an anonymous online survey. Midwives were chosen as study population because STS represents a serious professional risk in this population. In a series of confirmatory factor analyses and exploratory structural equation modeling (ESEM), a model with two correlated ESEM factors (i.e., intrusion, avoidance-arousal) provided the best model fit, thus establishing factorial validity. Differential associations of the STSS-F total score to general distress and posttraumatic stress and the utility of the STSS-F total score to account for variance in core dimensions of burnout beyond general distress, posttraumatic stress, perceived stress, occupational reward, and efforts supported the criterion validity of the STSS-F. The full STSS-F and its subscales showed acceptable to good levels of reliability. Limitations include the relatively small and homogeneous sample and the lack of tests of factorial invariance of the STSS-F and the original STSS. In conclusion, the present study provides evidence for the reliability and validity of the STSS-F. It makes the SSTS accessible to French speaking research contexts.

## Introduction

Secondary traumatic stress (*STS*) or STS disorder (*STSD*) is a syndrome including intrusion, avoidance, and arousal (2). The symptoms of STSD are the same as those of posttraumatic stress disorder (*PTSD*) in the fourth edition of the Diagnostic and Statistical Manual of Psychiatric Disorders [DSM-IV; (3)]. But unlike PTSD, STSD is due to indirect exposure in a professional context (e.g., caring for a traumatized patient)^1^. Hence, STSD was not included in DSM-IV as a formal psychiatric diagnosis. In the current DSM-5 (5), the new traumatic stressor criterion A4 identifies professional responsibilities as potential traumatic experiences that could precipitate PTSD. However, the DSM-5 disregards the helping and empathic quality of the relationship between primary and secondary traumatized victims. This reveals an important gap in the definitions of STSD in the DSM-5 and in the STS literature (6). The assignment of STSD to PTSD might also promote the misconception that STSD can easily be measured with standard PTSD inventories that usually do not refer to a specific traumatic event. But Renshaw et al. (7) showed that PTSD inventories likely provide an ambiguous measure of STS that may also tap into traumatic events experienced in respondents' own lives. Renshaw et al. (7) concluded that a more rigorous assessment of STS requires an explicit reference to indirect exposure. A recent publication setting out a research agenda for STSD highlighted that many previous studies have not made the important distinction between primary and secondary exposure to traumatic events and have called for the validation of screening tools for STS (8). A lack of conceptual clarity regarding the underlying constructs of STS has been discussed in the international literature. In particular, some authors use compassion fatigue interchangeably with STS, while others speak of compassion fatigue when describing a broad range of symptoms that include STS as well as burnout (8). The authors have therefore called for research that can provide evidence for the operationalization of STS and compassion fatigue that allow the development and validation of measures sensitive to the underlying concept (8).

In line with this reasoning, Bride et al. (1) developed the Secondary Traumatic Stress Scale (*STSS*), designed to measure the reactions of helping professionals who have experienced traumatic stress through their work with their traumatized clients. Consistent with the definition of PTSD in DSM-IV, STS is operationalized by the factors *intrusion, avoidance*, and *arousal* in the STSS. To enable a rigorous assessment of STS, the wording of the instruction and the stems of eight stressor-specific items refer explicitly to “client exposure” as traumatic stressor.

In the last decade, the STSS became a standard tool for assessing STS in helping professionals such as social workers (9), nurses (10), mental health workers (11), midwives (12), and pediatric care providers (13). In an international context, the STSS has been validated in Chilean professionals treating traumatized victims (14) and Italian ambulance workers (15). However, no French version of the STSS is available yet. Thus, the current paper aims to introduce the STSS-French version (*STSS-F*) and to evaluate its reliability, factorial and criterion validity and other psychometric properties in a sample of French speaking midwives in Switzerland.

Midwives are at risk of developing STS because they frequently have to manage traumatic births and other traumatic perinatal events (16, 17). A recent study of British midwives reported that over 95% of midwives had been directly or indirectly exposed to a work-related traumatic event (18). Recently, authors called for more studies investigating the structural nature of STS among different professional groups, as this seems to vary across professions (19). Given the aforementioned reasons, this study thus focused on one professional group: midwives.

Only a few studies have so far tested the factorial validity of the STSS [e.g., (1, 19–21)]. Those studies utilized the independent cluster model of confirmatory factor analysis (*ICM-CFA*), in which each item loads only on the factor it purports to measure and all cross-loadings are constrained to be zero (22). Given the limited evidence on the factorial validity of the STSS, we will test a series of five ICM-CFA models. These five models are based on prior research on the factorial structure of the STSS [e.g., (21)] and related DSM-IV-based PTSD instruments [e.g., (23)].

*Model 1* provides a test of the three correlated factors originally identified by Bride et al. (1) that parallel the PTSD symptom clusters in DSM-IV: Five intrusion items load on the intrusion factor, seven avoidance items load on the avoidance factor, and five arousal items load on the arousal factor. In prior research, this three-factorial ICM-CFA model fitted acceptably to the data [e.g., (1, 19–21)]. However, the factor correlation between avoidance and arousal often approaches unity [(19–21), (24) as cited in (21)] implying poor discriminant validity of the arousal and avoidance factors. Both factors might thus be pooled without substantial loss in model fit^2^. *Model 2* therefore tests the idea that an intrusion factor and a pooled avoidance-arousal factor are preferred over Model 1 due to parsimony and a comparable model fit. However, in the parallel DSM-IV based literature on PTSD instruments, little data support two-factor models (23). Model 2 also differs from alternative two-factor PTSD models that consist either of a re-experiencing/avoidance and a numbing/hyperarousal factor or of a depression/avoidance and an anxiety/ hyperarousal factor (25). In *Model 3*, a single STS factor will be specified. In Benuto et al. (19) and in Ting et al. (21), the χ^2^-difference test for Model 1 and Model 3 remained insignificant, suggesting that the unifactorial model might be a serious contender. But since the unifactorial model received no support in the parallel PTSD literature (23) and STSS intrusion usually correlates below .90 with avoidance and arousal (1, 20), a good fit for Model 3 seems questionable.

To the best of our knowledge, two four-factorial models derived from the DSM-IV based literature on PTSD still need to be tested in the context of the STSS: In *Model 4* [*numbing model*; (26)], the avoidance factor of Model 1 splits into narrower avoidance (two items) and emotional numbing (five items). This modification is justified by differential links of avoidance and numbing to external indices of treatment outcomes and psychopathology (27). *Model 5* [*dysphoria model*; (28)] retains the intrusion factor and King et al.'s (26) narrow avoidance factor. Three non-specific arousal items (criteria D1–D3) and five numbing items (criteria C3–C7) are assigned to the new dysphoria factor and two items (criteria D4 & D5) formed the hyperarousal factor. The dysphoria model takes into account that PTSD comprises a constellation of symptoms that reflect general emotional distress and dysphoria that may also be found in other anxiety and mood disorders (28). In the DSM-IV based PTSD literature, meta-analytical evidence suggests that both four-factor models outperform one- to three-factorial models and that Model 5 is slightly superior to Model 4 (23). Accordingly, it seems warranted to assume that in the context of the STSS, Model 5 may yield the best model fit of all five ICM-CFA models as well.

The Models 1–5 specify highly restrictive ICM-CFA models. However, items may have multiple determinants due to substantive theory (29), common method biases (30), or they may be fallible indicators of a factor (22). Items with small cross-loadings are thus frequently encountered in applied research (31). Imposing a perfect simple structure on such complex data leads to misspecified ICM-CFA models with impaired model fit and upwardly biased factor correlations (31). In prior studies on the STSS, the model fit of Model 1 mainly remained below the thresholds typically regarded as good fit and factor correlations were remarkably high [e.g., (1, 19–21)], suggesting the presence of misspecification in Model 1.

In the present study, we aimed to overcome the potential limitations inherent in Models 1–5 by using exploratory structural equation modeling (*ESEM*) (22, 31), which is, to the best of our knowledge, new in the context of the STSS. ESEM combines the strengths of exploratory factor analysis and confirmatory factor analysis. It allows for complex structure (i.e., items load on various factors) and for direct comparisons with the parametrically simpler ICM-CFA model nested within the more complex ESEM model (31). Given the nearly perfect correlations between avoidance and arousal in prior research [(19–21); see also Footnote 2], we expected that two well-defined correlated ESEM factors (i.e., intrusion and avoidance-arousal) will provide a good fit to the data (*Model 6*). We also expected that the two-factorial ESEM solution yields a better fit than the ICM-CFA Models 2, 4, and 5, and less correlated, more divergently valid factors than in Model 2.

The current study also aimed to provide preliminary evidence for the criterion validity of the STSS-F. First, PTSD as well as STSD include non-specific negative affect which gives rise to substantial positive associations between PTSD, STSD and general psychological distress [e.g., (1, 7, 28)]. However, given the close theoretical and empirical nexus between STSD and PTSD (2, 13), it was expected that the STSS-F total score relates positively and stronger to PTSD symptoms than to general psychological distress. Second, burnout consists of depersonalization, emotional exhaustion, and low personal accomplishments, which occur in response to chronic work-related stress (32). General emotional distress, posttraumatic stress, perceived stress, occupational efforts and rewards have been identified as reliable predictors or correlates of burnout [e.g., (33–37)]. Recent meta-analytical evidence suggests that STS is an important correlate of burnout as well (38). It was thus expected that the STSS-F total score will be related to the dimensions of burnout and that these relationships remain significant even when the effects of general psychological distress, posttraumatic stress, perceived stress, occupational rewards and efforts are statistically controlled for.

## Methods

### Participant Recruitment and Procedure

The study took place at two university hospitals in the French-speaking part of Switzerland. Midwives were chosen as study population because STS represents a serious professional risk in this population (12). All midwives working at both hospitals were eligible to participate in exchange for a paid extra hour of work to encourage their participation. They were informed about the study during staff meetings and by flyers and all were invited to participate. Staff accessing the anonymous online survey (LimeSurvey 2.0) found a detailed information sheet before giving informed consent. The survey consisted of seven questionnaires (one questionnaire is omitted in the current study) and took approximately 30 min to complete. All eligible participants received one reminder e-mail before the survey closed. Ethical approval was obtained from the ethics committee of the Canton de Vaud, Switzerland (study nr: 237/2013). Of the 280 eligible midwives, *N* = 220 participated (78.6% response rate).

### Measures

#### Secondary Traumatic Stress Scale (STSS) (1)

The STSS is a self-report inventory designed to assess the frequency of STS symptoms in professional caregivers. Respondents indicate on a 5-point Likert scale (1 = *never* to 5 = *very often*) how often they experienced each of the 17 STS symptoms during the last week. The wording of the instruction and eight items refer explicitly to client exposure as the traumatic stressor. The 17 items are organized in three subscales: intrusion, avoidance, and arousal. The STSS total score is calculated by summing up the item scores, with a higher score indicating a higher frequency of symptoms. A total score below 28 corresponds to “little or no STS,” a score between 28 and 37 means “mild STS,” between 38 and 43 “moderate STS,” between 44 and 48 “high STS,” and beyond 49 “severe STS” (9). In prior research, the STSS showed good psychometric properties (1, 9, 21). The STSS was translated into French using forward-backward translation and cultural adaptation (39).

#### Hospital Anxiety and Depression Scale—French Version (HADS) (40)

The HADS assesses anxiety and depression with two 7-item subscales. Each item is scored from 0 to 3, with higher scores indicating greater anxiety or depression. A recent meta-CFA revealed that a bi-factor structure with a strong general psychological distress factor and two small group factors reflecting depression and anxiety fits best to the HADS data (41). Norton et al. (41) concluded that the HADS may be appropriately used as a measure of general psychological distress. Hence, the current study drew on the HADS total score (ordinal Cronbach's α = 0.86; for descriptive statistics see Table 1).

**Table 1:** Descriptive statistics and Cronbach's α of study variables.

	***M***	**SD**	**α**
STSS-F Intrusion	9.16	3.63	0.84
STSS-F Avoidance	11.87	4.19	0.84
STSS-F Arousal	10.69	3.98	0.83
PSS total	35.85	7.98	0.89
PTSD-7	1.95	1.77	0.83
HADS Total	13.69	6.99	0.86
MBI Exhaustion	19.05	9.59	0.89
MBI Depersonalization	4.67	4.16	0.79
MBI Accomplishments	32.29	5.41	0.73
ERI Reward	2.77	0.53	0.88
ERI Effort	3.01	0.52	0.78

*STSS-F, Secondary Traumatic Stress Scale; PSS, Perceived Stress Scale; PTSD-7, PTSD screening scale; HADS, Hospital Anxiety and Depression Scale; MBI, Maslach Burnout Inventory; ERI, Effort Reward Imbalance Inventory. Coefficient alpha was calculated using either the polychoric or tetrachoric correlation matrix*.

#### Posttraumatic Stress Disorder (PTSD-7) (42)

The PTSD-7 is a short screening scale for the DSM-IV posttraumatic stress disorder (3). The PTSD-7 assesses five symptoms from the avoidance and numbing symptom cluster and two symptoms from the hyperarousal cluster using a dichotomous *yes/no* response format. In the instruction, these symptoms were referred to more generally as reactions that people sometimes have after a stressful event. A score of 4 or greater indicates positive cases of PTSD with a sensitivity of 80% and specificity of 97%. In the current study, ordinal coefficient α was good, α = 0.83.

#### Perceived Stress Scale-French Version (PSS) (43)

Perceived stress was measured with 14-items using a 5-point scale (1 = “*never*” to 5 = “*very often”*). Respondents indicated the degree to which they perceived their lives as overloaded and to which they appraised life events as unpredictable and uncontrollable during the last month. Correlations with other measures of objective or stress perception are positive, and adequate internal and re-test reliability have been reported (43). In the current study, the internal consistency of the PSS was good, ordinal α = 0.89.

#### Maslach Burnout Inventory—French Version (MBI) (44)

The MBI is a self-report measure designed to assess three core dimensions of burnout (32): *emotional exhaustion* (i.e., feeling emotionally overextended and exhausted by one's work; nine items), *depersonalization* (i.e., impersonal response toward recipients of one's service or care treatment; five items), and *personal accomplishment* (i.e., feeling competent and successful in one's work; eight items). Each items was rated on a 7-point scale (1 = “*never”* to 7 = “*every day”*). The French MBI evidenced good psychometric properties (44). In the current study, ordinal coefficients α of the subscales ranged from 0.73 to 0.89.

#### Effort-Reward Imbalance Questionnaire-French Version (ERI) (45)

The ERI is a self-report questionnaire developed to measure work related *effort* (six items; e.g., time pressure, interruptions, responsibility, working overtime, increasing demands), *reward* (10 items; e.g., money, esteem, career opportunities) and *over-commitment* (this scale was omitted in the present survey) using a 4-point scale (1 = “* strongly disagree”* to 4 = “*strongly agree”*). Effort and reward are used to calculate an effort/reward imbalance-ratio (45). However, the current study utilized both subscale scores directly as reliable indicators of occupational resources and stressors (reward: ordinal α = 0.88; effort: ordinal α = 0.78).

### Data Analysis

First, we elaborated the factorial validity of the STSS-F using a series of five ICM-CFAs: Model 1 consisted of the three factors originally identified by Bride et al. (1): intrusion (items 2, 3, 6, 10, 13), avoidance (items 1, 5, 7, 9, 12, 14, 17), and arousal (items 4, 8, 11, 15, 16). In Model 2, a pooled avoidance-arousal factor (12 items) was specified along with the intrusion factor. In Model 3, all 17 items loaded on a single STS factor. In Model 4 (numbing model), avoidance splits into narrow avoidance (items 12 and 14) and numbing (items 1, 5, 7, 9, and 17), leading to a four-factor model (intrusion, narrow avoidance, numbing, arousal). In Model 5 (dysphoria model), the narrow avoidance factor (items 12 and 14), a dysphoria factor (items 1, 4, 5, 7, 9, 11, 15, and 17), and a hyperarousal factor (items 8 and 16) were specified along with the intrusion factor. We also applied ESEM to the data (Model 6) (31). The number of factors to retain were determined via Velicer's (46) minimum average partial (*MAP*) test and via the Hull method based on the Common part Accounted For index (*Hull-CAF*) (47). The Hull-CAF aims to find the number of major factors that provides an optimal balance between number of parameters and model fit. The MAP test and Hull-CAF were based on polychoric correlations and they were carried out in FACTOR (48). The retained factors were target-rotated (22). Target rotation allows for more control on the expected factor structure. The target matrix was specified in a way that all intrusion items load on the expected intrusion factor, all avoidance and arousal items load on the expected avoidance-arousal factor and all estimated cross-loadings were targeted to be as close to zero as possible.

Factor analyses were carried out in Mplus 7 (49). Given that STSS scores are typically skewed (9) and that the rating scale might be regarded as five ordered categories, the data were modeled as ordinal using weighted least squares mean and variance-adjusted estimation (WLSMV). To evaluate the model fit, the conservative χ^2^-statistic of perfect fit was complemented by four fit indices: the normed χ^2^ (acceptable fit ≤ 3; good fit ≤ 2), the root mean square error of approximation (RMSEA; acceptable fit ≤ 0.08; good fit ≤ 0.06), the comparative fit index (CFI), and the Tucker Lewis Index (TLI; CFI & TLI: acceptable fit ≥0.90; good fit ≥0.95) (50). Nested models were compared by consistent results in the change χ^2^-test (DIFFTEST-option in Mplus) and practical relevant decrease in model fit [i.e., ΔTLI = TLI_constr._-TLI_unconstr._ < -0.01; (29)]. Predictive fit indices (e.g., AIC) are not available under WLSMV estimation, which precluded the comparison of non-nested models.

Second, in order to safeguard the usage of the STSS-F total score, item statistics and reliability coefficients (ordinal Cronbach's α and McDonald's ϖ_hierarchical_ and ϖ_total_) will be reported. Coefficients ϖ (51) were estimated with R 3.3.2 (52) and the Psych-package (53) based on minres-factoring and polychoric correlations.

Third, divergent and convergent validity of the STSS-F total und subscale scores was established by calculating zero-order and partial correlations with general distress and PTSD symptoms. Correlations were compared with Steiger's (54) *z* test for dependent correlations.

Finally, the utility of the STSS-F total score to account for variance in core dimensions of burnout beyond perceived stress, general distress, posttraumatic stress, and work related effort and reward was tested. The STSS-F total score entered first and the remaining variables entered at the second step. The first step tested the criterion validity of STSS-F and the second step probed the incremental validity of STSS-F (i.e., the test of STSS-F's partial effect is equivalent to the test of Δ*R*^2^ when the remaining mental health variables entered first and STSS-F total entered last). Standardized coefficients obtained for the STSS-F in the second step were compared with the coefficients of the remaining exogenous variables using the Wald test. The analyses were carried out in Mplus 7 based on *z*-scored variables and maximum likelihood estimation.

Due to *n* = 1 (HADS, PTSD-7) missing case, the effective sample sizes ranged from *N* = 219 to 220 in the analyses. Univariate outliers (*z*>3.29, *p* < 0.001) were found for STSS-F total and depersonalization. To reduce their impact in correlation and regression analyses, three scores (depersonalization) and one score (STSS-F total) were altered prior to the analyses [i.e., extreme raw scores were assigned values one unit larger than the next most extreme value in the distribution; for details see (55)].

## Results

### Establishing the Factorial Structure of the SSTS-F

The model fit statistics obtained in the six factor analyses are presented in Table 2, item loadings, variance explained and factor correlations for the Models 1, 2, 5, and 6 can be seen in Table 3. For all models, perfect fit to the data was rejected (for χ^2^-test results see Table 2). In terms of approximate fit, the three-factor model showed an acceptable fit to the data (see Table 2, Model 1). All items showed substantial item loadings (median, *Mdn* = 0.71, range: 0.54–0.79, all *p* < 0.05) and the explained item variances ranged from 29% to 63%. The high factor correlation between arousal and avoidance implied poor discriminant validity of both factors. The more restrictive Model 2 fitted acceptably to the data as well (see Table 2). Using the DIFFTEST-option in Mplus, Model 2 showed a significant drop in model fit when compared to Model 1, Δχ^2^(*df* = 2) = 6.34, *p* = 0.042. However, the small ΔTLI = −0.001 implied no practically relevant loss in model fit. Again, all items showed substantial item loadings (*Mdn* = 0.70, range: 0.53–0.79, all *p* < 0.05) and the levels of explained item variances remained fairly the same (range: 28–63%). The unifactorial solution (Model 3) failed to reach acceptable levels of model fit. Compared to Model 2, the more constrained Model 3 yielded a poorer model fit in terms of Δχ^2^(1) = 52.97, *p* < 0.001, and ΔTLI = −0.054. Thus, a single factor did not adequately account for the associations among the items. The test of Model 4 resulted in a non-positive definite PSI matrix, which rendered the solution uninterpretable. Model 5 reached the best fit indices of all ICM-CFA models (see Table 2). Compared to Model 2, Model 5 yielded a better fit in terms of χ^2^, Δχ^2^(*df* = 5) = 24.76, *p* < 0.001, but TLI change suggested a practically insignificant gain in model fit, ΔTLI = −0.008. Compared to Model 1 and 2, Model 5 reached slightly higher loadings (*Mdn* = 0.71, range: 0.56–0.85) and explained item variance (range: 31–73%) (see Table 3).

**Table 2:** Summary of model fit statistics from factor analyses of the STSS-F (*N* = 220).

**Models**	**χ^2^**	**df**	**χ^2^/df**	**CFI**	**TLI**	**RMSEA**	**90%-CI**
Model 1: Three correlated factors	242.30^***^	116	2.09	0.948	0.940	0.070	0.058–0.083
Model 2: Two correlated factors	246.72^***^	118	2.09	0.947	0.939	0.070	0.058–0.083
Model 3: One factor	364.80^***^	119	3.07	0.900	0.885	0.097	0.086–0.108
Model 4: Four correlated factors (King at al.)^a^	224.75^***^	113	1.99	0.954	0.945	0.067	0.054–0.080
Model 5: Four correlated factors (Simms et al.)	221.35^***^	113	1.96	0.956	0.947	0.066	0.053–0.079
Model 6: Two correlated ESEM factors	173.23^***^	103	1.68	0.971	0.962	0.056	0.041–0.070

*χ^2^, χ^2^ based on WLSMV estimation; df, degrees of freedom; χ^2^/df, normed χ^2^; CFI, comparative fit index; TLI, Tucker-Lewis index; RMSEA, root mean squared error of approximation and the respective 90%-CI; Model 1, three correlated factors [i.e., intrusion, avoidance, arousal; (1)]; Model 2, two correlated factors (i.e., intrusion, avoidance-arousal); Model 3, unidimensional model; Model 4, numbing model (26); Model 5, dysphoria model (28); Model 6, two target rotated ESEM factors (i.e., intrusion, avoidance-arousal); ^a^ inadmissible solution due to a non-positive definite PSI matrix*.

*** *p < 0.001*.

**Table 3:** Descriptive item statistics, factor loadings and factor correlations for four tested models (*N* = 220).

			**Model 1**				**Model 2**			**Model 5**					**Model 6**		
**STSS-F Items**	***M***	***SD***	**F_**1−1**_**	**F_**1−2**_**	**F_**1−3**_**	***R*^**2**^**	**F_**2−1**_**	**F_**2−2**_**	***R*^**2**^**	**F_**5−1**_**	**F_**5−2**_**	**F_**5−3**_**	**F_**5−4**_**	***R*^**2**^**	**F_**6−1**_**	**F_**6−2**_**	***R*^**2**^**
1. Felt emotionally numb	1.53	0.77		0.56		0.31		0.55	0.30			0.56		0.31	–0.17	0.69	0.38
2. Heart started pounding	1.87	1.04	0.79			0.63	0.79		0.62	0.79				0.62	0.65	0.15	0.56
3. Reliving client's trauma	1.54	0.78	0.74			0.55	0.74		0.55	0.74				0.55	0.67	0.10	0.53
4. Had trouble sleeping	2.57	1.33			0.58	0.33		0.58	0.34			0.59		0.35	0.29	0.37	0.33
5. Discouraged about future	2.19	1.20		0.71		0.51		0.70	0.49			0.72		0.52	0.21	0.55	0.48
6. Upset by reminders	2.04	1.00	0.65			0.42	0.65		0.43	0.65				0.42	0.66	0.03	0.45
7. Little interest	1.49	0.74		0.71		0.50		0.69	0.48			0.71		0.50	–0.22	0.88	0.61
8. Felt jumpy	1.90	1.02			0.70	0.49		0.70	0.49				0.71	0.50	0.07	0.66	0.50
9. Less active than usual	2.04	1.08		0.69		0.48		0.68	0.46			0.70		0.49	−0.07	0.75	0.51
10. Unintended thought	2.16	1.18	0.74			0.55	0.74		0.55	0.74				0.55	0.67	0.09	0.53
11. Trouble concentrating	2.11	1.08			0.78	0.61		0.79	0.62			0.80		0.64	−0.09	0.87	0.68
12. Avoid reminders	1.43	0.85		0.76		0.58		0.75	0.57		0.85			0.73	0.25	0.57	0.55
13. Disturbing dreams	1.55	0.94	0.63			0.40	0.63		0.40	0.63				0.40	0.76	−0.08	0.52
14. Avoid working with clients	1.55	0.81		0.54		0.29		0.53	0.28		0.59			0.35	0.14	0.43	0.27
15. Easily annoyed	2.36	1.04			0.72	0.52		0.73	0.53			0.74		0.55	0.13	0.64	0.52
16. Expected bad to happen	1.74	1.00			0.76	0.58		0.77	0.59				0.78	0.61	0.38	0.49	0.59
17. Memory gaps	1.65	0.87		0.62		0.38		0.61	0.37			0.62		0.39	−0.12	0.71	0.43
Factor correlations			0.66				0.70			0.72					0.56		
			0.75	0.99						0.63	0.84						
										0.80	0.77	0.94					

*Model 1, three correlated ICM-CFA factors (1); Model 2, two correlated ICM-CFA factors; Model 5, dysphoria model (28); Model 6, two target rotated ESEM factors; F_1-1_, F_2-1_, F_5-1_, F_6-1_, intrusion; F_2-2_, F_6-2_, avoidance-arousal; F_1-2_, avoidance; F_1-3_, arousal; F_5-2_, narrow avoidance; F_5-3_, dysphoria; F_5-4_, hyperarousal; R^2^, squared multiple correlation; ESEM loadings significant at p < 0.05 are underlined, secondary loadings |a > 0.30 are printed in italics*.

Both the MAP test and the Hull-CAF suggested two factors to retain. All four fit indices suggested a good fit for Model 6 (see Table 2). Compared to the ESEM model, the more constrained Model 2 yielded a poorer model fit (Model 2: Δχ^2^[*df* = 15] = 62.58, *p* < 0.001, and ΔTLI = −0.023). Model 5 is not nested under Model 6 which prevents a direct comparison of both models. However, Model 6 yielded better fit indices than Model 5 which is consistent with a better overall fit of Model 6^3^. Both ESEM factors jointly accounted for 27–68% of item variance (*Mdn* = 52%). Inspection of the factor loadings in Table 3 revealed that all primary loadings were on their expected factors, were statistically significant and, except for item 4 (*a* = 0.37), substantial in size (*Mdn* = 0.66; range: 0.37–0.88). Seven significant cross-loadings emerged. Except for item 16 (*a* = 0.38), all cross-loadings fell below a liberal standard of substantial loadings (i.e., |*a*| < 0.30), implying little or some influence of the respective factor on the construct relevant part of these indicators. Compared to Model 2, both ESEM factors were less correlated (*r* = 0.70 vs. *r* = 0.56) and demonstrated better discriminant validity. The correlated ESEM factor model is technically equivalent to a model including a substantial second-order STS factor (both second-order loadings constrained to be equal and estimated as *a* = sqrt[0.56] = 0.75)^4^, which bolsters the utility of the STSS-F total score.

### Descriptive Statistics and Reliability of STSS-F Variables

Means, standard deviations, and ordinal alpha of STSS-F variables were as follows: Full STSS-F (*M* = 31.71, *SD* = 10.09, range: 17-78, α = 0.92), intrusion (*M* = 9.16, *SD* = 3.63, range: 5–23, α = 0.84), and avoidance-arousal (*M* = 22.55, *SD* = 7.67, range: 12–57, α = 0.91) (for avoidance and arousal see Table 1). Using a cut-off score of 38 to determine the presence of STSD (9), *N* = 58 (26.4%) of the midwives were at or above the cut-off score suggesting a moderate prevalence rate of traumatization in the sample. The STSS-F total score was slightly skewed, skew = 0.78, and moderately kurtotic, kurtosis = 1.06 (intrusion: skew = 0.96, kurtosis = 0.72; avoidance-arousal: skew = 0.97, kurtosis = 1.30). At item level, skew ranged from 0.34 to 2.22 (*Mdn* = 0.98) and kurtosis ranged from −1.10 to 4.93 (*Mdn* = 0.27). The rate of endorsement to the response category “1 = *never”* ranged from 22.3% (item 15) to 74.5% (item 12) (*Mdn* = 50.5%; for 15 items, the mode was ‘1') which is comparable to the frequencies reported in Bride (9) for social workers. Item difficulties ranged from 11% (item 12) to 39% (item 4) with a median of 22%. All corrected item-total correlations of the 17 items in the STSS-F total score were satisfactorily (*Mdn* = 0.54, range: 0.40–0.66).

The coefficients omega estimated for the full STSS were ϖ_hierarchical_ = 0.77 and ϖ_total_ = 0.94. Omega total reflects a high degree of variance due to the major and group factors underlying the STSS-F, whereas ϖ_hierarchical_ indicates that a substantial proportion of item variance was due to a general STS factor. Thus, the STSS-F total score estimates a general STS factor that is common to all 17 STSS-F items at a satisfactory precision which provides additional support for the usage of the STSS-F total score in further analyses.

### Criterion Validity of the Full STSS-F

Pearson correlations and partial correlations including the STSS-F total, intrusion and avoidance-arousal subscales are shown Table 4. The STSS-F total score correlated positively with PTSD-7 and HADS total, but according to the *z*-test for dependent correlations, the correlation with PTSD-7 was significantly stronger, *z* = 3.22, *p* = 0.001. Intrusion was similarly related to general psychological distress and posttraumatic stress, which is likely due to the fact, that the PTSD-7 screener lacks intrusions. Accordingly, PTSD-7 was stronger related to avoidance-arousal than to intrusion, *z* = 7.02, *p* < 0.001. Avoidance-arousal was also positively related to HADS total, but this association was smaller compared to the link to posttraumatic stress, *z* = −4.42, *p* < 0.001. When HADS total was controlled for, the partial correlations between STSS-F total, avoidance-arousal and PTSD-7 remained strong, *pr* = 0.62, and *pr* = 0.67, *p* < 0.001, but the correlation with intrusion declined to *pr* = 0.27, *p* < 0.001. When PTSD-7 was controlled for, the associations between all three STSS-F variables and HADS total dropped but remained significant, implying weak to moderate specific associations between secondary traumatic stress and general psychological distress (*pr* = 0.27 to 0.37, all *p* < 0.001). In sum, the stronger overlap of STSS-F total with PTSD-7 than with HADS total, which becomes evident in the observed pattern of Pearson correlations and partial correlations, provides support for the criterion validity of the STSS-F.

**Table 4:** Pearson correlations (below the diagonal) and partial correlations (above the diagonal) between STSS-F variables and mental health variables.

	**STSS-F total^a^**	**Intrusion^a^**	**Avoidance-arousal^a^**	**PTSD-7**	**HADS total**
STSS-F total^a^	–	–	–	0.62^***^	0.37^***^
Intrusion^a^	0.77^***^^b^	–	–	0.27^***^	0.27^***^
Avoidance-arousal^a^	0.95^***^^b^	0.54^***^	–	0.67^***^	0.34^***^
PTSD-7	0.68^***^^c^	0.37^***^^d^	0.72^***^^d^, ^*e*^	–	0.03
HADS total	0.50^***^^c^, ^*f*^	0.37^***^	0.48^***^^e^	0.36^***^^f^	–

Partial correlations were either controlled for the respective STSS-F variable, PTSD-7, or HADS total;

a *Prior to the analyses one score was altered to reduce the impact of an univariate outlier*.

b ,

c ,

d ,

e ,

f Correlations with the same superscript differ in Steiger's (54) z-test for dependent correlations at p < 0.01;

*** *p < 0.001 (2-tailed); N = 219*.

When STSS-F total was regressed on general psychological distress, PTSD symptoms, perceived stress, occupational efforts, and rewards, a total of 60.6% of variance in STSS-F total was explained, *F*_(5, 213)_ = 65.54, *p* < 0.001. The strongest partial effect was found for PTSD-7, β = 0.44, *p* < 0.001, followed by perceived stress, β = 0.26, *p* < 0.001, HADS total, β = 0.12, *p* = 0.020, occupational reward, β = −0.11, *p* = 0.017, and effort, β = 0.10, *p* = 0.049. Thus, posttraumatic symptoms, and to a lesser extent perceived stress, general psychological distress, occupational efforts and rewards were all specifically and independently related to STS.

The results from regressing the core dimensions of burnout on STSS-F total and other mental health and work related variables are shown in Table 5. We also initially included the effort-reward imbalance ratio but since no significant partial effects for ERI-ratio were found in the presence of effort and reward, the ERI-ratio was dropped. When STSS-F total entered the model first, STS was related to depersonalization, β = 0.50, *p* < 0.001, exhaustion, β = 0.68, *p* < 0.001, and personal accomplishments, β = −0.39, *p* < 0.001, thereby supporting the criterion validity of the STSS-F. When the remaining variables entered the model in the second step, STSS-F total remained significantly related to depersonalization (β = 0.54, *p* < 0.001), exhaustion (β = 0.41, *p* < 0.001), and personal accomplishments (β = −0.18, *p* = 0.043). Thus, STS accounted for burnout variance beyond posttraumatic stress, general psychological distress, perceived stress, effort, and reward, which supports the incremental validity of the STSS-F. As indicated by the Wald test, STSS-F total was the strongest individual contributor to depersonalization (see Table 5). STSS-F total contributed also stronger to exhaustion than posttraumatic stress, perceived stress, and general distress. Thus, STS is highly relevant for both core dimensions of burnout.

**Table 5:** Hierarchical regression analyses with secondary traumatic stress entered at step 1, and posttraumatic stress, general distress, perceived stress, occupational effort, and rewards entered at step 2.

	**Depersonalization (MBI)^a^**			**Exhaustion (MBI)**			**Accomplishments (MBI)**		
	**β**	***SE***	***z***	**β**	***SE***	***z***	**β**	***SE***	***z***
1. Secondary traumatic stress (STSS-F)^a^	0.50	0.06	8.47^***^	0.68	0.05	13.61^***^	−0.39	0.06	−6.24^***^
2. Secondary traumatic stress (STSS-F)^a^	0.54	0.09	6.01^***^	0.41	0.07	5.95^***^	−0.18	0.09	−2.02^*^
Posttraumatic stress (PTSD-7)	−0.11^c^	0.08	−1.34	0.03^c^	0.06	0.45	−0.01	0.08	−0.12
General distress (HADS)	−0.01^c^	0.07	−0.16	−0.06^c^	0.05	−1.14	0.02	0.07	0.27
Perceived stress (PSS)	−0.07^c^	0.08	−0.86	0.14^b^	0.06	2.25^*^	−0.25	0.08	−3.11^**^
Effort (ERI)	0.23^b^	0.07	3.55^***^	0.25	0.05	5.00^***^	0.24^c^	0.07	3.73^***^
Reward (ERI)	0.04^c^	0.07	0.60	−0.24^c^	0.05	−4.84^***^	0.37^c^	0.06	5.72^***^
Step 1	*R*^2^ = 0.25, *SE* = 0.05*, z =* 4.88^***^			*R*^2^ = 0.46, *SE* = 0.05, *z* = 9.25^***^			*R*^2^ = 0.15, *SE* = 0.05, z = 3.39^***^		
Step 2	*R*^2^ = 0.30, *SE* = 0.05*, z =* 5.72^***^			*R*^2^ = 0.59, *SE* = 0.04, *z* = 14.03^***^			*R*^2^ = 0.31, *SE* = 0.05, *z* = 5.89^***^		

Regression analyses were carried out in Mplus using maximum likelihood estimation; β = standardized regression coefficient; SE, standard error; z, z-test (β/SE);

a Prior to the analyses one score (STSS-F total) and three scores (depersonalization) scores were altered;

b Coefficient differs at p < 0.01 from the respective STSS-F coefficient (Wald-test);

c Coefficient differs at p < 0.001 from the respective STSS-F coefficient (Wald-test); tolerance values for all predictor variables in the second step were ≥.49;

* p < 0.05;

** p < 0.01,

*** *p < 0.001; N = 219*.

## Discussion

The current study aimed to validate the French version of Bride et al.'s (1) Secondary Traumatic Stress Scale in a sample of *N* = 220 Swiss midwives by evaluating its factorial and criterion validity. Using the ICM-CFA approach (22), a series of five models was tested, of which the King et al. (26) numbing model, and the Simms et al. (28) dysphoria model were tested for the first time in the context of the STSS. A sixth model including two ESEM factors (31) was also estimated, which is also new in the context of the STSS. The more appropriate utilization of WLSMV estimation represents a methodological advance over recent studies that relied on normal theory estimators [e.g., (1, 19, 21)]. The results supported the factorial and concurrent validity, as well as the reliability of the STSS-F.

When testing the factorial validity, Bride et al.'s (1) three-factor model (Model 1) showed an acceptable model fit, suggesting configurational invariance for the original STSS and the STSS-F. Consistent to prior findings, intrusion and avoidance were poorly differentiated (20, 21). Thus, a more restrictive two-factor model including an intrusion factor and a pooled avoidance-arousal factor (Model 2) fitted acceptably to the data as well and showed no practically relevant loss in model fit (i.e., change in TLI). The single-factor model (Model 3) suggested by Benuto et al. (19) and Ting et al. (21) was not supported by the current data. This is hardly surprising, given that avoidance and arousal are usually differentiable from intrusion [(1, 20), (24) as cited in (21)]. However, Simms et al.'s (28) dysphoria model (Model 5) yielded the best fit of all ICM-CFA models [the test of the numbing model (26) resulted in an inadmissable solution]. Thus, rearranging avoidance and arousal symptoms into a dysphoria factor and two narrower avoidance and hyperarousal factors improved the model fit. This finding is in line with evidence on the factor structure of inventories assessing PTSD in accordance with DSM-IV's (3) PTSD criteria (23, 25).

Concluding that the factor structure of the STSS-F and of established PTSD inventories converge at the dysphoria model is premature in the light of the results obtained for Model 6. The ESEM analysis supported a two-factor model and both factors were readily interpretable as intrusion and avoidance-arousal [cf., (15)]. Model 6 is similar to the ICM-CFA Model 2, but it fitted significantly better than Model 2 and we found indications that it fitted also better to the data than Model 5. Consistent with the literature (22, 31), both ESEM factors were better differentiated than both factors in Model 2. The deflated factor correlation and the superior model fit of Model 6 were mainly due to seven small, yet statistically significant cross-loadings. These cross-loadings are consistent with the insight that in applied settings, psychometric indicators are seldom perfectly pure indicators of a given construct (31). However, only one cross-loading (item 16) can be considered to be of practical importance (i.e., *a* > 0.30) and this cross-loading is likely due to substantive theory: The expectation of “something bad to happen” mainly reflects arousal, but it also comprises the occurrence of future intrusions as “bad events.” This cross-loading thus reflects the influence of the intrusion factor on the construct-relevant part of item 16 and it allows intrusion to be estimated more precisely. In sum, the superior model fit and the better divergent validity of both ESEM factors imply that Model 6 provided the best representation of the factor structure underlying the STSS-F. It suggests that perfect simple structure does not adequately represent STSD phenomena. The marked preference of STSD-researchers for models that conform to perfect simple structure and their reliance on ICM-CFA models may have led to a biased understanding of the nature of STSD [for an exception see (15)]. However, covariation among STSD symptoms may also be under the influence of methodological factors (30). Thus, we need to emphasize that the two-factor structure may hold only for the STSS-F rather than for the STSD structure in general.

Interestingly, the two-factor model is inconsistent to prior research on the factor structure of PTSD instruments. As Elhai and Palmieri (25) note, little data support two-factor PTSD models that also diverge structurally from the current two-factor STSS model [e.g., intrusion/avoidance and numbing/hyperarousal factors; (56)]. However, the two-factor STSS model is consistent with prior findings of poorly differentiated arousal and avoidance ICM-CFA-factors [e.g., (19–21), see also (15)]. It thus cannot be dismissed as an artifact of item translation, sampling bias or a cultural idiosyncrasy. Instead, the present findings might indicate that the factor structures of the STSS and established PTSD instruments do not fully converge. If this holds true, this divergence might extend prior findings that the structure of PTSD symptom measures might be impacted by differences in trauma exposure [i.e., trauma-exposed vs. non-trauma exposed respondents; (25)]. However, recent attempts to bring a modified STSS into alignment with the DSM-5 (5) definition of PTSD supported a hybrid model, which connects fairly well to the DSM-5 based PTSD literature (6) and casts doubts on the potential divergence of the factorial structure of both STSD and PTSD symptoms. However, Mordeno et al. (6) did not test for an alternative ESEM model and their reliance on overly restrictive ICM-CFA models might have led to biased results.

Which conclusions regarding the factorial structure of the original STSS can be drawn from the current study? Consistent with the research on DSM-IV-based PTSD models (23), a single factor unlikely represents the factorial structure of the STSS. The three-factor model suggested by Bride et al. (1) regularly evidenced an acceptable model fit in prior research. The current results also suggest that the dysphoria model (28) is superior to the three-factor model and that both models are inferior to the two-factor ESEM model. The former suggestion is consistent with previous research on PTSD models (23). However, the two-factor ESEM model is parametrically more complex than both alternative models, which might impair its generalization. Given that tests of the factorial invariance of the STSS across different cultures and populations of helping professionals are lacking, it might be premature to discard the three-factor model and the dysphoria model in favor of the promising two-factor ESEM model. Thus, more research is needed to draw reliable conclusions on the factorial structure of the STSS in general and the two-factor ESEM model in particular. In order to investigate the structure of the STSS further, future research should consider Bayesian Structural Equation Modeling [BSEM; (57)]. Within BSEM, unimportant cross-loadings are specified to have a mean of zero and a small variance. As a consequence, BSEM with informative small-variance priors shrinks the cross-loadings toward their zero prior mean likely yielding smaller cross-loadings than ESEM with target rotation (57). This feature is appealing for a re-evaluation of the cross-loading obtained for item 16. Finally, once the factorial structure of the STSS has been sufficiently established, future research may improve the original STSS (1) and the DSM-5 adapted STSS (6) by applying item response theory [IRT; (58)]. IRT provides detailed information on individual item characteristics, which may help to improve the psychometric quality of the STSS by excluding poorly performing items.

In applied research settings, the STSS-F total score is usually taken as overall index of secondary traumatic stress. The present results also supported the criterion validity and reliability of the STSS-F total score: The correlated ESEM factors, which imply a substantial second-order STS factor, and the acceptable to good reliability of the STSS-F total score in terms of coefficient alpha, ϖ_hierarchical_, and ϖ_total_ support the usage of the STSS-F total score. The STSS-F total score was positively related to general psychological distress and PTSD symptoms, but the association with the latter was stronger, thus supporting the validity of the STSS-F. When PTSD symptoms were controlled for, the STSS-F total score remained positively related to general psychological distress. The specific associations of the full STSS-F with PTSD symptoms and general psychological distress suggest that the STSD symptoms have different etiologies in PTSD, depression, and anxiety. However, it has been recognized that PTSD and STSD include non-specific negative affect (28). Thus, these results are in line with the literature on PTSD and STSD and provide no threat to the validity of the STSS-F.

Finally, the core dimensions of burnout (35) were regressed on STSS-F total (step 1), mental health and occupation-related variables (step 2). In step 1, the full STSS-F was positively linked to emotional exhaustion and depersonalization and negatively linked to personal accomplishments. Given that STS is a reliable correlate of burnout (38), these findings bolster the criterion validity of the STSS-F. Perceived stress, general psychological distress, PTSD symptoms, occupational reward, and occupational effort accounted for 60.6% of variance in the STSS-F total score. And even when this substantial overlap was controlled for, the full STSS-F remained significantly related to all three core dimensions of burnout, thereby demonstrating incremental validity. We also compared the partial effects of the full STSS-F with the effects of the remaining exogenous variables in the regression model. For depersonalization, the strongest partial effect in the model was found for the full STSS-F. Moreover, the partial effect of the full STSS-F on exhaustion was stronger than the respective effects of posttraumatic stress, perceived stress, and general psychological distress. For personal accomplishments, the effect of the full STSS-F was less pronounced. These results provide further support for the notion that STS is of utmost importance for the understanding of burnout in helping professionals (38).

## Limitations

Several limitations of the current study need to be mentioned: First, the current sample size might be considered as rather small for factor analysis. However, WLSMV yields reliable test statistics, parameter estimates, and standard errors for medium sized CFA models and sample sizes as small as *N* = 200 (50). The ratio of cases per indicator in each model (1:13) and the sample size of *N* = 220 is sufficient for models with *df* ≥100 to achieve power of 0.80 for RMSEA-based tests of close fit (59). Thus, sample size unlikely represents a serious limitation to the current study. Second, no tests of factorial invariance of the STSS-F and the original STSS were conducted. As long as such tests are missing, the results obtained by both STSS versions need to be compared cautiously. Third, a homogeneous sample of midwives was used in the current study. Given that various variables may impact the factor structure of the STSS-F [see (25)] more research is needed to test the factorial invariance across various samples of helping professionals. Fourth, recent attempts have been made to bring the STSS in alignment with the DSM-5 definition of PTSD (6). However, this study was unavailable when the current study was conducted. Given that the original STSS is still in extensive use, we continued with the original STSS. Despite this, the modification of the STSS-F to assess STSD in line with DSM-5 is a necessary step in future research. A related limitation is the omission of symptoms of intrusion in the PTSD screening scale, which likely resulted in a downwardly biased association between PTSD symptoms and STSS-F intrusion. Fifth, the current data are cross-sectional, which prevents causal interpretations of the obtained effects. Sixth, the results are exclusively based on self-report data, which might be biased by several methodological factors (30). Finally, the present study was conducted as a web-based survey, which might cast doubt on the quality of the data. However, prior evidence suggests that traditional paper-and-pencil and web-based data collection methods yield basically equivalent data [e.g., (60, 61)].

Despite these limitations, the present study provides evidence for the validity and reliability of the STSS-F and thus makes the STSS accessible to French speaking research and clinical contexts. Future research might aim to compare different professional groups using the STSS-F.

## Author Contributions

IJ and AH conceived of and designed the study, jointly drafted the manuscript and approved the final version. IJ carried out the data analysis. IJ and AH contributed to the interpretation of data. MC and CM contributed to the design of the study, the data collection, and interpretation of the data. They also critically reviewed and approved the final draft of the manuscript. All authors agree to be accountable for all aspects of the work in ensuring that questions related to the accuracy or integrity of any part of the work are appropriately investigated and resolved.

### Conflict of Interest Statement

The authors declare that the research was conducted in the absence of any commercial or financial relationships that could be construed as a potential conflict of interest.

## Footnotes

^1^Although indirect exposure and secondary traumatization may occur within other contexts such as families or cultural systems as well [e.g., (4)], the current study is focused exclusively on work-related secondary traumatization (i.e.,client exposure as traumatic stressor).

^2^The Italian version of the STSS (15) suggests a similar structure consisting of an arousal factor (9 items) and an intrusion factor (6 items). However, it only contains 15 out of the original 17 STSS items.

^3^To substantiate this conclusion further we re-estimated both Models using robust maximum likelihood estimation (MLR). Model 6 yielded a lower Akaike Information Criterion, AIC = 9323.87, than Model 5, AIC = 9329.31, implying a better fit of Model 6 [cf. (50)].

^4^Strictly speaking, a 2-indicator factor is unidentified. But by either fixing both higher-order loadings to 1.0 or by fixing both loadings to be equal and fixing the second-order variance to 1.0, the higher-order part of the model becomes locally identified. However, testing the second-order part directly gains little additional insight because both fixed loadings are fully determined, and are a transformation of the factor correlation (loading *a* = sqrt[correlation]).

## References

[1] Bride BE Robinson MM Yegidis B Figley CR Development and validation of the secondary traumatic stress scale. Res Soc Work Pract. (2004) 14:27–35. 10.1177/1049731503254106

[2] Figley CR Carbonell JL Boscarino JA Chang J. A clinical demonstration model for assessing the effectiveness of therapeutic interventions: an expanded clinical trials methodology. Int J Emerg Ment Health. (1999) 1:155–64. 11232384

[3] American Psychiatric Association Diagnostic and Statistical Manual of Mental Disorders, 4th Edn, Text Revision. Washington, DC: American Psychiatric Publishing (2000).

[4] Figley CR Kiser LJ Helping Traumatized Families. (2012). New York, NY: Routledge.

[5] American Psychiatric Association Diagnostic and Statistical Manual of Mental Disorders, 5th Edn. Arlington, VA: American Psychiatric Publishing (2013).

[6] Mordeno IG Go GP Yangson-Serondo A. Examining the dimensional structure models of secondary traumatic stress based on DSM-5 symptoms. Asian J Psychiatr. (2017) 25:154–60. 10.1016/j.ajp.2016.10.024 28262139

[7] Renshaw KD Allen ES Rhoades GK Blais RK Markman HJ Stanley SM Distress in spouses of service members with symptoms of combat-related PTSD: secondary traumatic stress or general psychological distress? J Fam Psychol. (2011) 25:461 10.1037/a0023994 21639635 PMC3156850

[8] Molnar BE Sprang G Killian KD Gottfried R Emery V Bride BE Advancing science and practice for vicarious traumatization/secondary traumatic stress: a research agenda. Traumatology. (2017) 23:129 10.1037/trm0000122

[9] Bride BE. Prevalence of secondary traumatic stress among social workers. Soc Work. (2007) 52:63–70. 10.1093/sw/52.1.63 17388084

[10] Duffy E Avalos G Dowling M. Secondary traumatic stress among emergency nurses: a cross-sectional study. Int Emerg Nurs. (2015) 23:53–8. 10.1016/j.ienj.2014.05.001 24927978

[11] Creamer TL Liddle BJ. Secondary traumatic stress among disaster mental health workers responding to the September 11 attacks. J Traum Stress. (2005) 18:89–96. 10.1002/jts.20008 16281200

[12] Beck CT LoGiudice J Gable RK. A mixed-methods study of secondary traumatic stress in certified nurse-midwives: shaken belief in the birth process. J Midwifery Womens Health. (2015) 60:16–23. 10.1111/jmwh.12221 25644069

[13] Meadors P Lamson A Swanson M White M Sira N. Secondary traumatization in pediatric healthcare providers: compassion fatigue, burnout, and secondary traumatic stress. Omega (Westport). (2009) 60:103–28. 10.2190/OM.60.2.a 20222232

[14] Guerra C Saiz J Psychometric examination of the secondary traumatic stress scale: a study on chileans professionals. Psicol Conduct. (2007) 15:441–56.

[15] Setti I Argentero P Vicarious trauma: a contribution to the Italian adaptation of the secondary traumatic stress scale in a sample of ambulance operators. Bollettino di Psicologia Applicata. (2012) 264:58–64.

[16] Leinweber J Rowe HJ. The costs of 'being with the woman': secondary traumatic stress in midwifery. Midwifery. (2010) 26:76–87. 10.1016/j.midw.2008.04.003 18562056

[17] Sheen K Spiby H Slade P. Exposure to traumatic perinatal experiences and posttraumatic stress symptoms in midwives: prevalence and association with burnout. Int J Nurs Stud. (2015) 52:578–87. 10.1016/j.ijnurstu.2014.11.006 25561076

[18] Sheen K Slade P Spiby H. An integrative review of the impact of indirect trauma exposure in health professionals and potential issues of salience for midwives. J Adv Nurs. (2014) 70:729–43. 10.1111/jan.12274 24118130

[19] Benuto LT Yang Y Ahrendt A Cummings C The secondary traumatic stress scale: confirmatory factor analyses with a national sample of victim advocates. J Interpers Violence. (2018) 1:0886260518759657 10.1177/0886260518759657 29528798

[20] Mirsaleh YR Ahmadi K Davoudi F Mousavi SZ Validity, reliability, and factor structure of secondary trauma stress scale (STSS) in a sample of warfare victims' children. Iran J Psychiatry Clin Psychol. (2014) 20:134–43. Available online at: http://ijpcp.iums.ac.ir/article-1-2186-en.html

[21] Ting L Jacobson JM Sanders S Bride BE Harrington D The secondary traumatic stress scale (STSS) confirmatory factor analyses with a national sample of mental health social workers. J Hum Behav Soc Environ. (2005) 11:177–94. 10.1300/J137v11n03_09

[22] Marsh HW Morin AJ Parker PD Kaur G. Exploratory structural equation modeling: an integration of the best features of exploratory and confirmatory factor analysis. Annu Rev Clin Psychol. (2014) 10:85–110. 10.1146/annurev-clinpsy-032813-153700 24313568

[23] Yufik T Simms LJ. A meta-analytic investigation of the structure of posttraumatic stress disorder symptoms. J Abnorm Psychol. (2010) 119:764–76. 10.1037/a0020981 21090877 PMC4229035

[24] Bride BE Psychometric Properties of the Secondary Traumatic Stress Scale. Unpublished doctoral dissertation, University of Georgia, Athens (2001).

[25] Elhai JD Palmieri PA. The factor structure of posttraumatic stress disorder: a literature update, critique of methodology, and agenda for future research. J Anxiety Disord. (2011) 25:849–54. 10.1016/j.janxdis.2011.04.007 21793239

[26] King DW Leskin G King LA Weathers F Confirmatory factor analysis of the clinician-administered PTSD scale: evidence for the dimensionality of posttraumatic stress disorder. Psychol Assess. (1998) 10:90–6. 10.1037/1040-3590.10.2.90

[27] Asmundson GJ Stapleton JA Taylor S. Are avoidance and numbing distinct PTSD symptom clusters? J Trauma Stress. (2004) 17:467–75. 10.1007/s10960-004-5795-7 15730065

[28] Simms LJ Watson D Doebbeling BN. Confirmatory factor analyses of posttraumatic stress symptoms in deployed and nondeployed veterans of the Gulf War. J Abnorm Psychol. (2002) 111:637–47. 10.1037/0021-843X.111.4.637 12428777

[29] Gignac GE Multi-factor modeling in individual differences research: some recommendations and suggestions. Person Indiv Diff. (2007) 42:37–48. 10.1016/j.paid.2006.06.019

[30] Podsakoff PM MacKenzie SB Lee JY Podsakoff NP. Common method biases in behavioral research: a critical review of the literature and recommended remedies. J Appl Psychol. (2003) 88:879–903. 10.1037/0021-9010.88.5.879 14516251

[31] Asparouhov T Muthén B Exploratory structural equation modeling. Struct Equ Model A Multidisciplin J. (2009) 16:397–438. 10.1080/10705510903008204

[32] Maslach C Jackson SE The measurement of experienced burnout. J Organ Behav. (1981) 2:99–113. 10.1002/job.4030020205

[33] Alarcon GM A meta-analysis of burnout with job demands, resources, and attitudes. J Vocat Behav. (2011) 79:549–62. 10.1016/j.jvb.2011.03.007

[34] Khamisa N Peltzer K Oldenburg B. Burnout in relation to specific contributing factors and health outcomes among nurses: a systematic review. Int J Environ Res Pub Health. (2013) 10:2214–40. 10.3390/ijerph10062214 23727902 PMC3717733

[35] Maslach C Schaufeli WB Leiter MP. Job burnout. Ann Rev Psychol. (2001) 52:397–422. 10.1146/annurev.psych.52.1.397 11148311

[36] Xie Z Wang A Chen B. Nurse burnout and its association with occupational stress in a cross-sectional study in Shanghai. J Adv Nurs. (2011) 67:1537–46. 10.1111/j.1365-2648.2010.05576.x 21261698

[37] Yu X Wang P Zhai X Dai H Yang Q The effect of work stress on job burnout among teachers: the mediating role of self-efficacy. Soc Indicat Res. (2015) 122:701–8. 10.1007/s11205-014-0716-5

[38] Cieslak R Shoji K Douglas A Melville E Luszczynska A Benight CC. A meta-analysis of the relationship between job burnout and secondary traumatic stress among workers with indirect exposure to trauma. Psychol Serv. (2014) 11:75–86. 10.1037/a0033798 23937082

[39] Wild D Grove A Martin M Eremenco S McElroy S Verjee-Lorenz A. Principles of good practice for the translation and cultural adaptation process for patient-reported outcomes (PRO) measures: report of the ISPOR task force for translation and cultural adaptation. Value in Health. (2005) 8:94–104. 10.1111/j.1524-4733.2005.04054.x 15804318

[40] Bocerean C Dupret E. A validation study of the hospital anxiety and depression scale (HADS) in a large sample of French employees. BMC Psychiatry. (2014) 14:354. 10.1186/s12888-014-0354-0 25511175 PMC4476305

[41] Norton S Cosco T Doyle F Done J Sacker A. The hospital anxiety and depression scale: a meta confirmatory factor analysis. J Psychosom Res. (2013) 74:74–81. 10.1016/j.jpsychores.2012.10.010 23272992

[42] Breslau N Peterson EL Kessler RC Schultz LR. Short screening scale for DSM-IV posttraumatic stress disorder. Am J Psychiatry. (1999) 156:908–11. 10.1176/ajp.156.6.908 10360131

[43] Cohen S Kamarck T Mermelstein R. A global measure of perceived stress. J Health Soc Behav. (1983) 24:385–96. 10.2307/2136404 6668417

[44] Dion G Tessier R Validation de la traduction de l'lnventaire d'epuisement professionnel de Maslach et Jackson. Revue canadienne des sciences du comportement. (1994) 26:210–27. 10.1037/0008-400X.26.2.210

[45] Siegrist J Starke D Chandola T Godin I Marmot M Niedhammer I. The measurement of effort-reward imbalance at work: European comparisons. Soc Sci Med. (2004) 58:1483–99. 10.1016/S0277-9536(03)00351-4 14759692

[46] Velicer WF Determining the number of components from the matrix of partial correlations. Psychometrika. (1976) 41:321–7. 10.1007/BF02293557

[47] Lorenzo-Seva U Timmerman ME Kiers HA. The Hull method for selecting the number of common factors. Multivariate Behav Res. (2011) 46:340–64. 10.1080/00273171.2011.564527 26741331

[48] Ferrando PJ Lorenzo-Seva U. Program FACTOR at 10: Origins, development and future directions. Psicothema. (2017) 29:236–40. 10.7334/psicothema2016.304 28438248

[49] Muthen L Muthen B Mplus Version 7 User's Guide. Version 7: Muthen & Muthen (2012).

[50] Brown TA Confirmatory Factor Analysis for Applied Research. New York, NY: Guilford (2006).

[51] McDonald RP Test Theory: A Unified Approach. Mahwah, NJ: Lawrence (1999).

[52] R Core Team. R: A Language and Environment for Statistical Computing. Vienna: R Foundation for Statistical Computing (2016). Available online at: http://www.R-project.org/

[53] Revelle W Procedures for Personality and Psychological Research. (2016). Available online at: http://cran.r-project.org/web/packages/psych/R packageversion 1.6.4 (accessed April, 2016).

[54] Steiger JH Tests for comparing elements of a correlation matrix. Psychol Bull. (1980) 87:245–51. 10.1037/0033-2909.87.2.245

[55] Tabachnkick BG Fidell LS Using Multivariate Statistics, Sixth Edition, Pearson New International Edition. Harlow, UK: Pearson Education Limited (2014).

[56] Taylor S Kuch K Koch WJ Crockett DJ Passey G. The structure of posttraumatic stress symptoms. J Abnorm Psychol. (1998) 107:154–60. 10.1037/0021-843X.107.1.154 9505048

[57] Muthén B Asparouhov T. Bayesian structural equation modeling: a more flexible representation of substantive theory. Psychol Methods. (2012) 17:313–35. 10.1037/a0026802 22962886

[58] Embretson SE Reise SP Item Response Theory for Psychologists. New York, NY: Psychological Press (2000).

[59] MacCallum RC Browne MW Sugawara HM Power analysis and determination of sample size for covariance structure modeling. Psychol Methods. (1996) 1:130–49. 10.1037/1082-989X.1.2.130

[60] Davidov E Depner F Testing for measurement equivalence of human values across online and paper-and-pencil surveys. Qual Quant. (2011) 45:375–90. 10.1007/s11135-009-9297-9

[61] Weigold A Weigold IK Russell EJ. Examination of the equivalence of self-report survey-based paper-and-pencil and internet data collection methods. Psychol Methods. (2013) 18:53–70. 10.1037/a0031607 23477606

